# Activity-Based Contracting for Insecticide-Treated Net (ITN) Mass Distribution Campaigns in the Democratic Republic of Congo: A Multi-province Quasi-experimental Evaluation

**DOI:** 10.7759/cureus.117895

**Published:** 2026-10-14

**Authors:** Joris L Likwela, Michele K Luntadila, Eric M Tsasa, Albert N Kalonji, Adrien K N'siala, Philippe N Lukanu

**Affiliations:** 1 Public Health, Université de Kisangani, Kisangani, COD; 2 Technical Direction, Soins de Santé Primaires en Milieu Rural (SANRU), Kinshasa, COD; 3 Global Fund/Long-Lasting Insecticidal Nets (GF/LLIN) Mass Campaign Project, Soins de Santé Primaires en Milieu Rural (SANRU), Kinshasa, COD; 4 Global Fund/Long-Lasting Insecticidal Nets (GF/LLIN) Mass Campaign Project, Interchurch Medical Assistance World Health, Kinshasa, COD; 5 Family Medicine, Université Protestante au Congo, Kinshasa, COD

**Keywords:** activity-based contracting, democratic republic of congo, insecticide-treated mosquito net, malaria, mass campaign, results-based financing

## Abstract

Background

Operational inefficiencies continue to limit the effectiveness of insecticide-treated net (ITN) mass distribution campaigns, particularly in challenging operating environments such as the Democratic Republic of the Congo (DRC). Results-based contracting has been proposed to improve campaign performance, but evidence regarding its effectiveness at scale remains limited.

Materials and methods

We conducted a quasi-experimental evaluation of ITN mass distribution campaigns implemented in 20 provinces of the DRC between 2022 and 2024, comparing four provinces applying an activity-based contracting (ABC) approach with 16 provinces applying the standard input-based approach. The primary outcome was compliance with ITN allocation norms, as verified by independent agencies. Secondary outcomes included independently verified household coverage, campaign preparation time, and unit cost per ITN distributed. Three comparison frameworks were combined: concurrent, within-province historical, and contextually matched. Because the ABC approach was assigned at the provincial level, we analyzed all indicators at both the household level (two-sample Z-tests for proportions with risk differences and 95% confidence intervals) and the provincial level (exact Wilcoxon rank-sum tests, with the province as the unit of observation).

Results

Compliance with ITN allocation norms was higher in ABC provinces than in non-ABC provinces (94.4% versus 85.6%; risk difference +8.8 percentage points, 95% CI +8.6 to +9.0). The difference persisted when province was the unit of analysis, with complete separation between the two groups: the four ABC provinces occupied the four highest ranks among the 20 (median 93.6% versus 85.6%; exact p = 0.0004). It was reproduced in the matched four-versus-four comparison (exact p = 0.029). All secondary outcomes were more favorable in ABC provinces: independently verified household coverage 98.6% versus 95.8%, adjusted campaign preparation time 14.6 versus 22.8 weeks, and unit cost USD 0.668 versus USD 0.773 per ITN distributed, but none reached statistical significance at the provincial level, and all are reported as exploratory. Measured ITN loss rates were higher under ABC (0.33% versus 0.17%); because losses were established by independent physical inventory under ABC and by administrative balance elsewhere, the two figures are not measurement-equivalent and were not compared statistically.

Conclusions

Scaling up ABC beyond a single pilot province was associated with higher compliance with ITN allocation norms, consistently across concurrent, historical, and matched comparison frameworks and at the level at which the intervention was assigned. Differences in coverage, timelines, and unit costs pointed in the same direction but were not statistically established. Results-based contracting merits consideration to improve the quality of large-scale distribution campaigns in resource-constrained settings, pending confirmation from evaluations conducted independently of program implementers and using designs that allow formal control of confounding.

## Introduction

Malaria remains a major global public health challenge, with an estimated 282 million cases and nearly 610,000 deaths worldwide in 2024, the vast majority of which occurred in sub-Saharan Africa. Five countries, namely Nigeria (24.3%), the Democratic Republic of the Congo (DRC) (12.5%), Uganda (4.7%), Ethiopia (4.4%), and Mozambique (3.6%), accounted for nearly half of all malaria cases worldwide that year [1]. Despite substantial progress over the past two decades, recent trends indicate stagnation or reversal in several high-burden countries, particularly those facing persistent structural and operational constraints.

The World Health Organization Global Malaria Program has defined four strategic priorities for 2024-2030: norms and standards, innovation, strategic information for impact, and leadership [2]. With constrained resources and a global malaria funding gap of USD 3.7 billion, innovation increasingly requires improving the efficiency and delivery of existing tools rather than relying solely on new technologies [3,4]. Vector control through insecticide-treated nets (ITNs) remains a cornerstone of malaria prevention, and ITN mass distribution campaigns are the most effective way to rapidly increase household ownership and population-level coverage [5,6]. However, since around 2015, gains in ITN access and use have plateaued, suggesting that expanding nominal coverage alone may be insufficient without improvements in campaign quality, compliance, and operational efficiency [4].

This plateau is largely explained by persistent operational constraints affecting mass distribution campaigns: inaccurate population data leading to suboptimal planning, recipients’ tendency to overstate household size, which can result in stock-outs; disruptions caused by emerging epidemics such as COVID-19 and mpox; and limited campaign effectiveness associated with weak leadership and low community ownership [7-9].

In the DRC, these challenges are compounded by complex implementation environments and stringent donor requirements attached to grants from the Global Fund to Fight AIDS, Tuberculosis, and Malaria (Global Fund). While essential for fiduciary accountability, input-based financing mechanisms that emphasize compliance with procurement and financial management procedures have historically contributed to prolonged delays, increased transaction costs, and reduced attention to supervision and data quality [10]. Despite these controls, audits have continued to identify recurring inefficiencies, highlighting the limits of input-based financing in ensuring the effective, timely delivery of malaria interventions [10].

In response, results-based financing approaches, and specifically results-based contracting, have gained attention in challenging operating environments [11]. In the DRC, this approach was operationalized as activity-based contracting (ABC), under which payments to health zones are conditional on independent verification of predefined performance indicators relating to ITN campaign delivery, household coverage, compliance with allocation norms, and stock management [12]. A pilot implementation of the ABC model in Kwilu Province suggested improvements in coverage quality, implementation timelines, and accountability compared with the standard input-based approach [12].

Unlike facility-level performance-based financing, which ties payment to routine service indicators, ABC applies to a time-bound mass distribution campaign coordinated at the health zone level, where the deliverable is a single, independently verifiable event; payment is therefore tied to campaign-specific indicators (household coverage, allocation compliance, and stock reconciliation) rather than to routine service volumes [12].

A critical evidence gap remains. It is not known whether the improvements observed in the Kwilu pilot are reproducible at scale across provincial contexts that differ substantially in population density, logistical infrastructure, and security conditions. Pilot implementations of contractual mechanisms are typically supported by concentrated technical assistance, close supervision, and a degree of institutional attention that cannot be sustained when a model is extended; whether gains observed in a single, closely supervised province persist under routine implementation is therefore an empirical question distinct from the one the pilot answered. To our knowledge, no study has examined whether extending the ABC model beyond a single pilot province yields consistent improvements across the operational indicators that determine campaign efficiency and quality.

This study addresses this gap. Its primary objective was to compare compliance with ITN allocation norms between four provinces applying ABC and 16 provinces applying the standard approach, using routine program data from ITN mass distribution campaigns implemented in 20 provinces between 2022 and 2024. Its secondary objectives were to compare independently verified household coverage, campaign preparation time, and unit cost per ITN distributed and to describe measured ITN loss rates. Given the non-randomized design, this study assesses the associations between the contracting approach and campaign performance and does not seek to establish causation.

We hypothesized that provinces implementing the ABC approach would achieve higher compliance with ITN allocation norms than provinces implementing the standard approach. Compliance was designated the primary outcome because it is the indicator most directly tied to contractual payment and therefore the one on which an incentive mechanism would be expected to act first. We examined secondary and exploratory questions to determine whether the approach was also associated with higher independently verified household coverage, shorter campaign preparation time, and lower unit costs. We did not assume all indicators would move in the same direction; in particular, we anticipated that measured ITN loss rates might be higher under ABC because of more stringent physical verification rather than poorer stock management.

## Materials and methods

Scope of the study

The study covers ITN mass distribution campaigns implemented in 20 DRC provinces between 2022 and 2024 with Global Fund financing. Sixteen provinces applied the standard input-based approach: Bas-Uélé, Equateur, Haut-Katanga, Haut-Lomami, Ituri, Kinshasa, Kongo Central, Lomami, Mai-Ndombe, Maniema, North Kivu, Sankuru, South Kivu, Tanganyika, Tshopo, and Tshuapa. Four provinces applied the ABC approach: Kwilu, Haut-Uélé, Mongala, and Sud-Ubangi (Figure 1).

**Figure 1:**
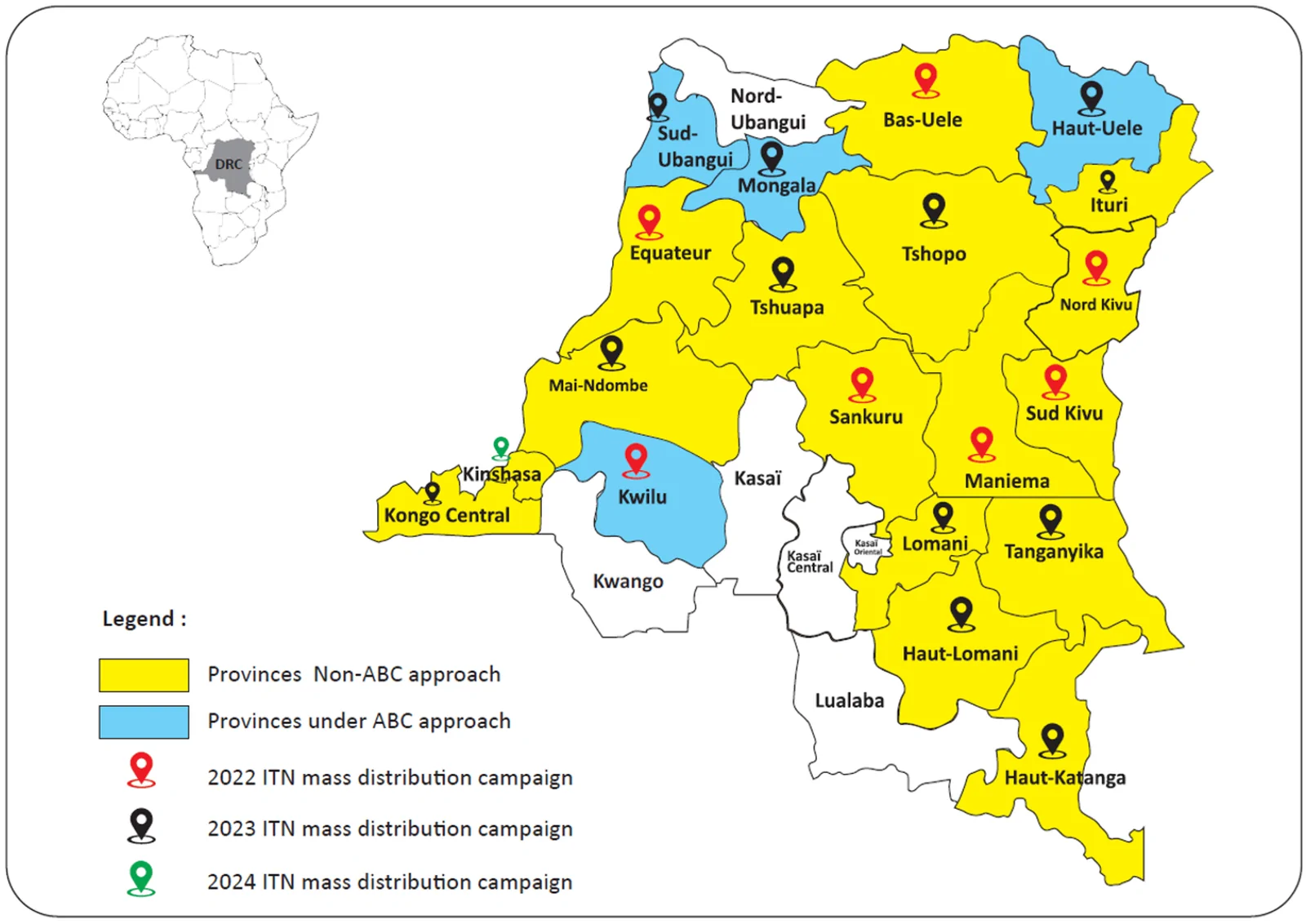
Mapping of ITN mass distribution campaigns implemented from 2022 to 2024 Provinces shaded in blue used the ABC approach, whereas those shaded in yellow used the standard input-based approach. Markers indicate the campaign year. ITN: insecticide-treated net, ABC: activity-based contracting. Image credit: Created by the SANRU team using Adobe Illustrator (Adobe Inc., San Jose, California, USA).

Provincial context and selection of ABC provinces

The 20 provinces included in this evaluation differed substantially in population density, security conditions, and logistical accessibility, as reflected in target populations, household counts, and recorded logistical delays. These contextual dimensions were addressed by design, through the matching of ABC provinces to non-ABC provinces of common administrative origin (see comparative analysis, below), rather than through direct statistical adjustment. The authors did not have province-level data on malaria endemicity or indicators of health system capacity (staffing density, facility infrastructure) for inclusion in this evaluation; they acknowledge this absence as a limitation affecting the generalizability of the findings, as discussed in the Limitations section.

The selection of provinces for the ABC approach was not randomized. Kwilu Province hosted the initial pilot [12]. The National Malaria Control Program later decided to extend the study to three additional provinces: Mongala, Sud-Ubangi, and Haut-Uélé. SANRU provided technical and financial support to Sud-Ubangi and Haut-Uélé through its sub-recipient, Interchurch Medical Assistance World Health. The authors are not aware of a documented or published selection protocol. They cannot rule out that logistical readiness, partner presence, donor considerations, or the perception that some provinces were more likely to succeed under a contractual mechanism influenced which provinces were selected. This constitutes a specific selection-bias risk, addressed further in the Limitations section.

Study design

A quasi-experimental design was selected as the most appropriate framework given the programmatic context. The assignment of the ABC approach to provinces was based on operational and programmatic considerations, not randomization. This is a characteristic of large-scale public health program evaluations in real-world settings, where randomization is neither feasible nor ethical. In the taxonomy of non-randomized evaluations, this study corresponds to a non-equivalent control group design with an added pretest.

In the absence of randomization, three complementary comparison frameworks were combined: a concurrent comparison between the four ABC provinces and the 16 non-ABC provinces operating within the same national campaign cycle and funding environment; within-province historical comparisons between each ABC province and its performance during the preceding campaign conducted under the standard approach; and a comparison restricted to ABC provinces and non-ABC provinces matched on contextual similarity. Convergence across these frameworks is treated as the principal basis for inference, as no single framework is sufficient on its own.

Outcomes

Compliance with ITN allocation norms, defined as the proportion of visited households that received the number of ITNs corresponding to the number of people living in the household, as established by independent verification agencies, was the primary outcome. It is the indicator most directly tied to contractual payment under the ABC approach, counting towards 75% of the total price payable to health zones [12], and therefore the outcome on which a results-based mechanism would be expected to act first.

Independently verified household coverage, campaign preparation time, and unit cost per ITN distributed were designated as secondary outcomes. The ITN loss rate and the administratively reported coverage ratio were treated as descriptive process indicators for the reasons set out under Statistical Analysis.

Data sources, sampling, and data quality

Data came from four standardized programmatic sources: health zone micro-planning reports; distribution databases completed in real time by community health workers on digital devices; provincial data validation workshop reports; and independent verification agency reports. Figure 2 shows the main stages of the ITN mass campaign (Panel A), the chronology of the stages producing the data used to calculate health zone performance indicators (Panel B), and the chronology of the independent verifications (Panel C).

**Figure 2:**
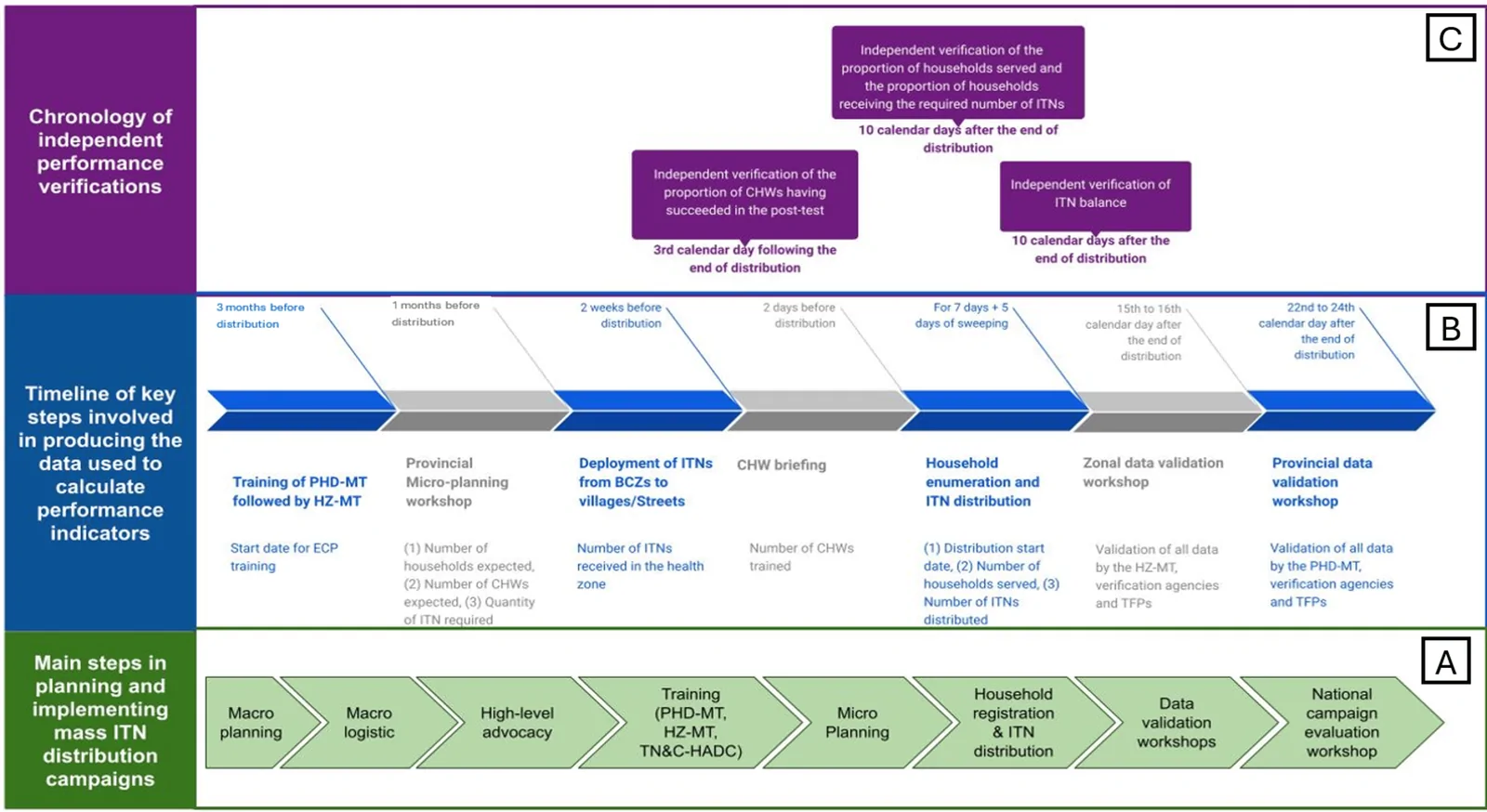
Timelines of key steps in ITN mass distribution campaigns PHD-MT: Provincial Health Division Management Team, HZ-MT: Health Zone Management Team, ITN: insecticide-treated nets, HZCO: Health Zone Central Office, CHW: community health worker, TN: titular nurse, C-HDAC: Chairperson, Health Area Development Committee. Image credit: Created by the SANRU team using the Google Drawings web app (Google LLC, Mountain View, California, USA).

Sampling was applied to the independent verification component only; all other indicators were derived from exhaustive programmatic records. Verification surveys followed the standardized protocol described for the Kwilu pilot [12]. Within each health zone, households were selected by systematic random sampling from the distribution lists, with a sampling interval derived from the number of households served. Across the 20 provinces, verification agencies visited 595,383 households.

The SANRU server applied automated, real-time consistency checks to transmitted data, flagging implausible household sizes, probable “ghost households,” and programmatic discrepancies; flagged records were referred to health zone and provincial supervisors for correction during the campaign. Records that remained inconsistent at the close of the provincial data validation workshop were excluded from the final database. The routine campaign information system does not retain an audit trail of these exclusions; therefore, their volume cannot be quantified retrospectively. No imputation was performed for missing data, and indicators for which the underlying data were unavailable are reported as not available in the tables.

Changes to the ABC approach since the Kwilu pilot

The contractual and operational arrangements described in our earlier report on Kwilu Province remain unchanged, except for the advance disbursement schedule shown in Figure 3.

**Figure 3:**
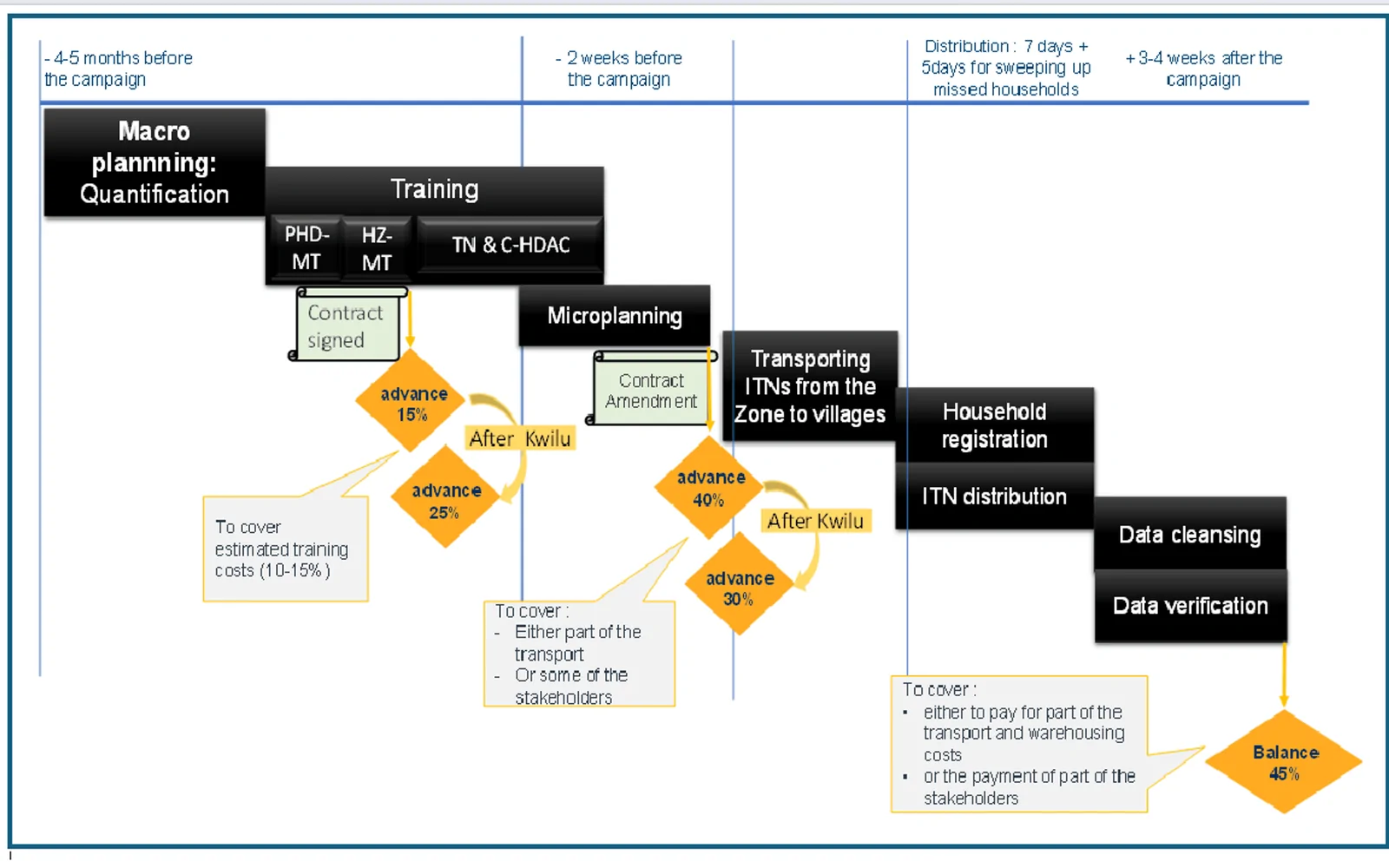
Contracting and payment terms of the ABC contract PHD-MT: Provincial Health Division Management Team, HZ-MT: Health Zone Management Team, ITN: insecticide-treated net, TN: titular nurse, C-HDAC: Chairperson, Health Area Development Committee Image credit: Created by the SANRU team using Microsoft PowerPoint (Microsoft Corp., Redmond, Washington, USA).

Advances are released against contractual milestones, with the final balance conditional on the independent verification of performance.

Indicators

The rationale for the contractual performance indicators used to determine payments to health zones under the ABC approach has been described previously [12]. The contract used three indicators: the proportion of households covered, the proportion of community health workers passing the post-training test, and the ITN loss rate. For the present comparison, two of these were retained, as the third was verified only in ABC provinces.

Campaign stages were identical in all provinces, except for an additional stage in ABC provinces: the contract between Interchurch Medical Assistance World Health, a sub-recipient of SANRU, and the health zones. Contracting took place at the end of the health zone management team training. Independent verification of household coverage and ITN balances was conducted in both ABC and non-ABC provinces.

The proportion of households covered was calculated as the number of households provided with ITNs divided by the number of households visited by verifiers. Health zones calculated their own coverage as the number of households provided with ITNs divided by the number of micro-planned households. This second quantity exceeded 100% in six provinces. Its denominator is a projection derived from administrative population estimates rather than an enumeration and is systematically underestimated where population growth or displacement has outpaced projections. It is therefore a ratio, not a proportion; throughout this report, it is designated the administrative coverage ratio, presented descriptively, and not subjected to tests for proportions or confidence interval (CI) calculation. The verification agency calculated the ITN loss rate as the number of ITNs lost divided by the number of ITNs received in the health zone.

Campaign preparation time was measured as the number of days elapsed between the start of training of the Provincial Health Division Management Team, which coordinates health interventions in the province, and the launch of ITN distribution to households, expressed in weeks. An adjusted campaign preparation time was also calculated by deducting time lost to logistical constraints independent of the contracting approach, defined as delays in provinces where ITNs arrived more than four weeks after the close of the micro-planning workshop, the threshold recommended in national malaria program standards.

Costs were derived from the validated budgets of health zone micro-plans, which constituted the payment basis for all expenditure related to ITN distribution to households; penalties charged to health zones under the ABC approach were added to those budgets. The penalty schedule has been described previously [12]. Because budgets grow with population, unit costs were obtained by dividing each provincial budget by the number of ITNs distributed in that province.

Comparative analysis

Each of the five indicators was compared across the three frameworks. The concurrent framework compared the four ABC provinces with the 16 non-ABC provinces within the same Global Fund allocation cycle. The historical framework compared each ABC province with its own preceding campaign, conducted two to four years earlier under the standard input-based approach and financed under the previous allocation cycle. The matched framework compared the four ABC provinces with four non-ABC provinces selected for contextual similarity.

Matching used the country’s administrative redistricting as the basis for contextual similarity, on the reasoning that provinces created from the same parent province share geography, population structure, and logistical profile. Kwilu (ABC) and Mai-Ndombe (non-ABC) both derive from the former Bandundu Province. Mongala and Sud-Ubangi (ABC) and Equateur and Tshuapa (non-ABC) all derive from the former Equateur Province. Haut-Uélé (ABC), Bas-Uélé, and Tshopo (non-ABC) all derive from the former Orientale Province; Tshopo was selected over Bas-Uélé so as not to weight the comparison in favor of the ABC group on population size.

The cost comparison was restricted to the historical framework and to Sud-Ubangi and Haut-Uélé, the two ABC provinces receiving technical and financial support from SANRU through its sub-recipient. Kwilu and Mongala were excluded because their preceding campaigns predated the COVID-19 pandemic, which substantially altered campaign design and precluded reasonable comparison [9].

Statistical analysis

The analyses were conducted at two levels. At the household level, comparisons between ABC and non-ABC provinces were conducted using two-sample Z-tests for proportions [13], with absolute risk differences and 95% CIs calculated by Newcombe’s method (based on Wilson score intervals), and risk ratios with 95% CIs (log method) reported alongside. Group proportions were pooled over all households sampled in the provinces of each group.

Because the ABC approach was assigned at the provincial level while the household was the unit of these calculations, the resulting test statistics overstate the precision of the comparisons; they are reported as descriptive measures of the magnitude of the observed differences and not as the inferential basis of the study.

At the provincial level, each province contributes a single observation, and the four ABC provinces are compared with the 16 non-ABC provinces using an exact Wilcoxon rank-sum test. This analysis is conducted at the level at which the intervention was actually assigned and is free of the unit-of-analysis assumption underlying the Z-tests. In a four-versus-16 configuration, the smallest attainable two-sided p-value is 0.0004; in the four-versus-four matched comparison, it is 0.029. This analysis serves as the sensitivity analysis for every indicator, and the findings are considered robust when the two levels agree.

Two indicators were withdrawn from hypothesis testing. The administrative coverage ratio is not a proportion, for the reason set out above. ITN loss rates were established by independent physical inventory in ABC provinces and by administrative balance in non-ABC provinces, because the two measures are not equivalent; comparing them by a test of proportions would attribute to the contracting approach a difference that is at least partly a difference in measurement. Both are reported descriptively, with the measurement differential stated explicitly.

No correction for multiple comparisons was applied across the five indicators. Compliance was the single primary outcome, and the remaining indicators were secondary or descriptive and carried no independent inferential claim. No multivariable adjustment for confounding was performed, as the aggregated nature of the available provincial-level data precluded regression modeling at the health zone or household level. With individual-level household data, generalized estimating equations or mixed-effects (multilevel) logistic regression, with health zone or province specified as a clustering or random-effects term, would have been the preferred approach to account for within-province correlation directly; however, the household-level records underlying the aggregated provincial counts used in this evaluation were not available to the authors in a form permitting such modeling. The provincial-level exact Wilcoxon analysis described above was adopted as the feasible alternative: by making the province, rather than the household, the unit of observation, it removes the within-province correlation problem that motivates clustering-robust methods, at the cost of the statistical power such methods would otherwise preserve. The design also addressed confounding through study design rather than statistical analysis: matching on administrative origin was intended to improve comparability in geography, population structure, and logistical access by construction, although it cannot adjust for security conditions or the intensity of partner supervision, which remain unadjusted. Multivariable logistic regression, fixed-effects models, and difference-in-differences analysis would have permitted more rigorous control of confounding but could not be applied to aggregated data. Findings are accordingly interpreted as programmatic associations rather than established causal effects.

Analyses were performed using Stata 16 (StataCorp LLC, College Station, TX, USA) [14], using the prtesti command for two-sample tests of proportions and the ranksum command with the exact option for provincial-level comparisons (exact randomization distribution, tied values receiving averaged ranks). Statistical significance was assessed at the 5% level (α = 0.05).

Ethical considerations

This study retrospectively evaluated a national public health program using aggregated routine programmatic data. Under Article 69 of Law No. 18/035 of 13 December 2018 establishing the fundamental principles governing the organization of public health in the DRC, biomedical research on a human subject is defined as activity directed at the human person, at organs, tissues, cells, or other products derived from the human body, or at personal information files. The present evaluation involved none of these: no primary data collection was undertaken for research purposes, no personal information file was used, and the indicators analyzed are counts aggregated at the health zone and provincial level. It therefore falls outside the scope of Article 69 and outside the approval regime established by Articles 63 and 73 of the same Law. Accordingly, ethics committee approval was not required, and individual informed consent was not applicable. The independent verification surveys from which household denominators were derived were conducted as a contractual component of program implementation, under protocols described previously [12]. The data used are routine program data held by SANRU, to which the authors have access through their program monitoring roles.

## Results

Between 2022 and 2024, 20 ITN mass distribution campaigns were conducted in the DRC, co-financed by the Global Fund and the Against Malaria Foundation. The 20 provinces received 58,722,796 ITNs, for an estimated target population of 117,825,450.

Household coverage and compliance with allocation norms

Table 1 presents household coverage for each of the 20 provinces, as verified by independent civil society agencies, and compliance with ITN allocation norms.

**Table 1:** Household ITN coverage and compliance with allocation norms by province and contracting approach (2022-2024) Verified household coverage is the number of households provided with ITNs divided by the number of households visited by the verification agencies. Compliance with ITN allocation norms is the proportion of visited households that received the number of ITNs corresponding to their household size. Summary rows are pooled across the households sampled in the provinces of each group. ITN: insecticide-treated net, ABC: activity-based contracting

N°	Province	Year	Approach	Households sampled	Verified household coverage (%)	Compliance with ITN allocation norms (%)
1	Bas-Uélé	2022	Non-ABC	14,100	92	84
2	North Kivu	2022	Non-ABC	24,026	93.2	88.5
3	South Kivu	2022	Non-ABC	25,208	95.6	86
4	Equateur	2022	Non-ABC	23,182	95.2	82.4
5	Maniema	2022	Non-ABC	29,187	96	88.5
6	Sankuru	2022	Non-ABC	22,717	98.4	91.3
7	Mai-Ndombe	2023	Non-ABC	24,239	96	86
8	Lomami	2023	Non-ABC	13,983	92.1	82.8
9	Tshuapa	2023	Non-ABC	23,042	99.9	85.1
10	Haut-Katanga	2023	Non-ABC	29,314	99.8	79.8
11	Haut-Lomami	2023	Non-ABC	44,183	97.9	81.1
12	Tanganyika	2023	Non-ABC	11,403	95.5	77
13	Kongo Central	2023	Non-ABC	52,536	98.1	91.5
14	Tshopo	2023	Non-ABC	39,812	98	75.1
15	Ituri	2023	Non-ABC	52,160	96.1	88.5
16	Kinshasa	2024	Non-ABC	106,247	92	88
All non-ABC provinces			Non-ABC	535,339	95.8	85.6
17	Kwilu	2022	ABC	16,033	99.9	98.9
18	Mongala	2023	ABC	15,209	97.5	92.1
19	Sud-Ubangi	2023	ABC	18,134	98.3	91.8
20	Haut-Uélé	2023	ABC	10,668	98.6	95.1
All ABC provinces			ABC	60,044	98.6	94.4

Compliance with ITN allocation norms, the primary outcome, was 94.4% in ABC provinces and 85.6% in non-ABC provinces, a difference of 8.8 percentage points (95% CI +8.6 to +9.0; risk ratio 1.103, 95% CI 1.100 to 1.105). Independently verified household coverage was 98.6% and 95.8%, respectively, a 2.8 percentage-point difference (95% CI +2.7 to +2.9; risk ratio 1.029, 95% CI 1.028 to 1.030). The administrative coverage ratio was 95.5% and 93.3%, respectively, and is reported descriptively only (Table 2).

**Table 2:** Administrative coverage ratio by province and contracting approach (2022-2024) The administrative coverage ratio is the number of households served divided by the number of households projected during micro-planning. Values above 100% occur when the number of households served exceeds the projected number, reflecting an underestimation of the denominator in administrative population projections rather than overdistribution. This measure is a ratio, not a proportion, and is reported for descriptive purposes only. ABC: activity-based contracting

N°	Province	Year	Approach	Micro-planned households	Households served	Administrative coverage ratio (%)
1	Bas-Uélé	2022	Non-ABC	293,194	303,668	103.6
2	North Kivu	2022	Non-ABC	1,955,627	1,744,098	89.2
3	South Kivu	2022	Non-ABC	1,697,648	1,555,822	91.6
4	Equateur	2022	Non-ABC	623,719	530,083	85
5	Maniema	2022	Non-ABC	631,723	595,172	94.2
6	Sankuru	2022	Non-ABC	503,848	475,344	94.3
7	Mai-Ndombe	2023	Non-ABC	642,749	440,895	68.6
8	Lomami	2023	Non-ABC	909,044	911,378	100.3
9	Tshuapa	2023	Non-ABC	493,833	421,504	85.4
10	Haut-Katanga	2023	Non-ABC	1,742,528	1,569,666	90.1
11	Haut-Lomami	2023	Non-ABC	860,473	868,144	100.9
12	Tanganyika	2023	Non-ABC	868,500	770,352	88.7
13	Kongo Central	2023	Non-ABC	1,333,476	1,250,569	93.8
14	Tshopo	2023	Non-ABC	797,497	818,895	102.7
15	Ituri	2023	Non-ABC	1,127,844	1,351,462	119.8
16	Kinshasa	2024	Non-ABC	2,533,860	2,266,102	89.4
All non-ABC provinces			Non-ABC	17,015,563	15,873,154	93.3
17	Kwilu	2022	ABC	1,254,257	1,157,980	92.3
18	Mongala	2023	ABC	692,947	599,658	86.5
19	Sud-Ubangi	2023	ABC	658,360	632,432	96.1
20	Haut-Uélé	2023	ABC	416,716	496,341	119.1
All ABC provinces			ABC	3,022,280	2,886,411	95.5

ITN loss rates

Table 3 compares ITN loss rates between campaigns implemented under the ABC approach and those conducted under the standard input-based approach, using aggregated health zone data on ITNs received, distributed, remaining, and reported as lost.

**Table 3:** ITN loss rates by province and contracting approach (2022-2024) Loss rates in ABC provinces were derived from an independent physical inventory of remaining ITNs, whereas those in non-ABC provinces were derived from the administrative balance between ITNs received and ITNs reported as distributed. These measures are not equivalent and are therefore reported descriptively. ITN: insecticide-treated net, ABC: activity-based contracting

N°	Province	Year	Approach	ITNs received	ITNs distributed	ITN balance	ITNs lost	Loss rate (%)
1	Bas-Uélé	2022	Non-ABC	836,718	821,063	10,543	5,112	0.61
2	North Kivu	2022	Non-ABC	6,017,556	5,910,351	106,952	253	<0.01
3	South Kivu	2022	Non-ABC	5,340,260	5,083,525	221,353	35,382	0.66
4	Equateur	2022	Non-ABC	1,663,000	1,648,509	12,461	2,030	0.12
5	Maniema	2022	Non-ABC	1,928,662	1,807,121	100,033	21,507	1.12
6	Sankuru	2022	Non-ABC	1,570,832	1,525,533	44,131	1,168	0.07
7	Mai-Ndombe	2023	Non-ABC	1,309,720	1,275,767	33,747	206	0.02
8	Lomami	2023	Non-ABC	2,874,990	2,753,905	120,279	806	0.03
9	Tshuapa	2023	Non-ABC	1,340,466	1,303,311	35,982	1,173	0.09
10	Haut-Katanga	2023	Non-ABC	4,946,977	4,896,632	46,881	3,464	0.07
11	Haut-Lomami	2023	Non-ABC	2,658,237	2,577,150	80,813	274	0.01
12	Tanganyika	2023	Non-ABC	2,253,553	2,225,871	25,637	2,045	0.09
13	Kongo Central	2023	Non-ABC	3,159,647	2,934,853	223,963	831	0.03
14	Tshopo	2023	Non-ABC	2,442,540	2,346,074	90,397	6,069	0.25
15	Ituri	2023	Non-ABC	4,111,536	4,037,545	73,387	604	0.01
16	Kinshasa	2024	Non-ABC	7,273,447	7,198,442	73,381	1,624	0.02
All non-ABC provinces			Non-ABC	49,728,141	48,345,652	1,299,940	82,548	0.17
17	Kwilu	2022	ABC	3,703,408	3,563,582	127,624	12,202	0.33
18	Mongala	2023	ABC	1,910,459	1,895,044	10,366	5,049	0.26
19	Sud-Ubangi	2023	ABC	1,983,841	1,959,003	17,863	6,975	0.35
20	Haut-Uélé	2023	ABC	1,396,947	1,349,929	41,816	5,202	0.37
All ABC provinces			ABC	8,994,655	8,767,558	197,672	29,428	0.33

The average measured loss rate was 0.33% in ABC campaigns and 0.17% in non-ABC campaigns. The gap narrows when ABC provinces are compared with their preceding campaigns (0.33% versus 0.27%).

Campaign preparation time

Table 4 presents the interval between the start of Provincial Health Division Management Team training and the launch of household distribution, before and after adjustment for logistical delays, independent of the contracting approach.

**Table 4:** Campaign preparation time by province and contracting approach (2022-2024) Time lost to logistics was calculated for provinces where ITNs arrived more than four weeks after the micro-planning workshop closed, the threshold recommended by national malaria program standards. Delays resulted from carrier underperformance in South Kivu, Lomami, Mongala, and Sud-Ubangi, waiting for digitalization handsets in Maniema, and ITN redeployment following micro-planning in Sud-Ubangi. Dates are given in day/month/year format. ABC: activity-based contracting, ITN: insecticide-treated net

N°	Province	Year	Approach	Training start	Campaign launch	Elapsed (days)	Elapsed (weeks)	Time lost to logistics (weeks)	Adjusted time (weeks)
1	Bas-Uélé	2022	Non-ABC	16/08/2021	08/03/2022	204	29.1	0	29.1
2	North Kivu	2022	Non-ABC	30/10/2021	28/06/2022	241	34.4	0	34.4
3	South Kivu	2022	Non-ABC	16/07/2021	22/08/2022	402	57.4	13	44.4
4	Equateur	2022	Non-ABC	08/07/2022	28/11/2022	143	20.4	0	20.4
5	Maniema	2022	Non-ABC	05/08/2021	08/06/2022	307	43.9	5	38.9
6	Sankuru	2022	Non-ABC	12/10/2021	30/05/2022	230	32.9	0	32.9
7	Mai-Ndombe	2023	Non-ABC	15/02/2023	20/05/2023	94	13.4	0	13.4
8	Lomami	2023	Non-ABC	14/05/2022	02/12/2022	202	28.9	13	15.9
9	Tshuapa	2023	Non-ABC	10/09/2022	15/01/2023	127	18.1	0	18.1
10	Haut-Katanga	2023	Non-ABC	02/01/2023	25/04/2023	113	16.1	0	16.1
11	Haut-Lomami	2023	Non-ABC	11/06/2023	25/10/2023	136	19.4	0	19.4
12	Tanganyika	2023	Non-ABC	22/02/2023	22/07/2023	150	21.4	0	21.4
13	Kongo Central	2023	Non-ABC	23/08/2023	07/12/2023	106	15.1	0	15.1
14	Tshopo	2023	Non-ABC	03/07/2023	11/11/2023	131	18.7	0	18.7
15	Ituri	2023	Non-ABC	12/09/2023	13/12/2023	92	13.1	0	13.1
16	Kinshasa	2024	Non-ABC	10/10/2023	13/01/2024	95	13.6	0	13.6
Mean, non-ABC provinces			Non-ABC			173	24.7	1.9	22.8
17	Kwilu	2022	ABC	15/04/2022	29/06/2022	75	10.7	0	10.7
18	Mongala	2023	ABC	15/10/2022	24/03/2023	160	22.9	10	12.9
19	Sud-Ubangi	2023	ABC	01/02/2023	26/09/2023	237	33.9	15	18.9
20	Haut-Uélé	2023	ABC	19/06/2023	10/10/2023	113	16.1	0	16.1
Mean, ABC provinces			ABC			146	20.9	6.3	14.6

Unadjusted, ABC campaigns were on average 3.9 weeks shorter than non-ABC campaigns. After deducting time lost to logistical constraints independent of the contracting approach, the adjusted mean preparation times were 14.6 weeks for ABC campaigns and 22.8 weeks for non-ABC campaigns, a difference of 8.2 weeks.

Historical framework

When comparing the performance of ABC provinces with their performance in preceding campaigns under the standard approach, independently verified household coverage was available for comparison in only two provinces, Sud-Ubangi and Haut-Uélé, where verification data exist for both campaigns; it was essentially unchanged (98.4% versus 98.4%). Compliance with ITN allocation norms was markedly higher during the ABC campaign in Haut-Uélé (95.1% versus 77.3%) (Tables 1-5). The historical comparison for compliance rests on that single province, since compliance was not documented in the earlier campaigns of the three other ABC provinces; it is reported for completeness and should be read accordingly.

**Table 5:** Performance of the four ABC provinces during their preceding campaigns under the standard approach Compliance with ITN allocation norms was documented in only one of the four preceding campaigns; therefore, the subtotal for this indicator reflects Haut-Uélé alone. Independent verification was not conducted for the preceding campaigns in Kwilu and Mongala. The pooled verified coverage of the preceding campaigns (98.4%) includes only Sud-Ubangi and Haut-Uélé. ABC: activity-based contracting, ITN: insecticide-treated net

N°	Province	Year	Approach	Households sampled	Verified coverage (%)	Compliance with ITN allocation norms (%)	ITNs received	ITNs lost	Loss rate (%)	Adjusted campaign preparation time (weeks)
1	Kwilu	2018	Non-ABC	Not available	Not available	Not available	2,944,827	12,784	0.43	17.6
2	Mongala	2019	Non-ABC	Not available	Not available	Not available	1,586,578	5,697	0.36	17.9
3	Sud-Ubangi	2020	Non-ABC	28,371	97.3	Not available	1,832,550	1,018	0.06	26.4
4	Haut-Uélé	2021	Non-ABC	20,675	100	77.3	1,131,667	914	0.08	20.3
All four provinces			Non-ABC	49,046	98.4	77.3	7,495,622	20,413	0.27	20.6

Across the four ABC provinces, the pooled ITN loss rate was 0.33% (29,428 of 8,994,655 ITNs received), compared with 0.27% (20,413 of 7,495,622) in the same provinces during their preceding campaigns under the standard approach, a difference of 0.05 percentage points; the direction was not uniform, with lower rates in Kwilu (0.33% versus 0.43%) and Mongala (0.26% versus 0.36%), and higher rates in Sud-Ubangi (0.35% versus 0.06%) and Haut-Uélé (0.37% versus 0.08%) (Tables 3-5).

Compared with their own preceding campaigns under the standard approach, the four ABC provinces had a mean preparation time of 20.9 weeks versus 22.4 weeks (1.5 weeks shorter) and a mean adjusted campaign preparation time of 14.6 versus 20.6 weeks (5.9 weeks shorter). The adjusted time was shorter in all four provinces (by 6.9 weeks in Kwilu, 5.0 weeks in Mongala, 7.5 weeks in Sud-Ubangi, and 4.2 weeks in Haut-Uélé). In contrast, the unadjusted time was shorter in Kwilu and Haut-Uélé, unchanged in Sud-Ubangi and longer in Mongala (22.9 versus 17.9 weeks) (Tables 4-5).

Campaign costs

Table 6 compares the validated health zone micro-plan budgets of Sud-Ubangi and Haut-Uélé under the ABC approach with those of their preceding campaigns under the standard approach.

**Table 6:** Validated health zone micro-plan budgets under the ABC approach compared with the preceding campaign under the standard approach (Sud-Ubangi and Haut-Uélé) Provincial and partner support covers activities carried out by the Provincial Health Division and implementation support partners. Costs exclude the operation of the independent verification mechanism, which is financed separately by the principal recipient. Cost per ITN is calculated by dividing the total cost by the number of ITNs distributed. ABC: activity-based contracting, ITN: insecticide-treated net

Province and cost scope	Non-ABC total cost (USD)	Non-ABC cost per ITN (USD)	ABC total cost (USD)	ABC cost per ITN (USD)	Change in cost per ITN (USD)
Sud-Ubangi, health zone activities	1,216,860.74	0.690	1,222,541.28	0.620	−0.070
Sud-Ubangi, including provincial and partner support	1,300,377.92	0.730	1,249,305.28	0.640	−0.090
Haut-Uélé, health zone activities	859,715.73	0.760	946,049.84	0.701	−0.059
Haut-Uélé, including provincial and partner support	959,442.17	0.848	962,675.84	0.713	−0.135
Both provinces, health zone activities	2,076,576.46	0.716	2,168,591.12	0.655	−0.061
Both provinces, including provincial and partner support	2,243,051.48	0.773	2,211,981.12	0.668	−0.105

Under the standard approach, campaigns in Sud-Ubangi and Haut-Uélé cost USD 2,243,051.48 to distribute 2,900,295 ITNs, or USD 0.773 per ITN. Under the ABC approach, campaigns in the same two provinces two to three years later cost USD 2,211,981.12 to distribute 3,308,932 ITNs, or USD 0.668 per ITN, a reduction of USD 0.105 per ITN distributed.

Matched comparison

Table 7 compares the four ABC campaigns with four non-ABC campaigns selected for contextual similarity based on common administrative origin.

**Table 7:** Selected performance indicators in four ABC campaigns and four contextually matched non-ABC campaigns Following the country's redistricting, provinces were matched based on their shared administrative origins: Kwilu with Mai-Ndombe (former Bandundu Province), Mongala and Sud-Ubangi with Equateur and Tshuapa (former Equateur Province), and Haut-Uélé with Tshopo (former Orientale Province). ABC: activity-based contracting, ITN: insecticide-treated net

N°	Province	Year	Approach	Households sampled	Verified coverage (%)	Compliance with ITN allocation norms (%)	ITNs received	ITNs lost	Loss rate (%)	Adjusted campaign preparation time (weeks)
1	Equateur	2022	Non-ABC	23,182	95.2	82.4	1,663,000	2,030	0.12	20.4
2	Mai-Ndombe	2023	Non-ABC	24,239	96.0	86.0	1,309,720	206	0.02	13.4
3	Tshuapa	2023	Non-ABC	23,042	99.9	85.1	1,340,466	1,173	0.09	18.1
4	Tshopo	2023	Non-ABC	39,812	98.0	75.1	2,442,540	6,069	0.25	18.7
Matched non-ABC campaigns			Non-ABC	110,275	97.4	81.1	6,755,726	9,478	0.14	17.7
5	Kwilu	2022	ABC	16,033	99.9	98.9	3,703,408	12,202	0.33	10.7
6	Mongala	2023	ABC	15,209	97.5	92.1	1,910,459	5,049	0.26	12.9
7	Sud-Ubangi	2023	ABC	18,134	98.3	91.8	1,983,841	6,975	0.35	18.9
8	Haut-Uélé	2023	ABC	10,668	98.6	95.1	1,396,947	5,202	0.37	16.1
ABC campaigns			ABC	60,044	98.6	94.4	8,994,655	29,428	0.33	14.6

In the matched comparison, compliance with ITN allocation norms was 94.4% under ABC and 81.1% under the standard approach, a 13.2-percentage-point difference. Verified household coverage was 98.6% and 97.4%, respectively, a 1.2-percentage-point difference. Adjusted campaign preparation time averaged 14.6 weeks in ABC campaigns and 17.7 weeks in the matched non-ABC campaigns, a difference of 3.0 weeks; without adjustment for logistical constraints, the ABC campaigns were 3.2 weeks longer. Measured loss rates were 0.33% and 0.14%, respectively, subject to the measurement caveat noted above.

Provincial-level analysis

Because the ABC approach was assigned at the provincial level, all indicators were reanalyzed with the province as the unit of observation (Table 8).

**Table 8:** Comparison of campaign performance indicators between ABC and non-ABC provinces at household level and at provincial level Group proportions are pooled across the sampled households. Exact p-values are based on the exact randomization distribution, with averaged ranks for tied values. Household-level risk differences were calculated using Newcombe’s method (based on Wilson score intervals) and are reported only for the two indicators that are true proportions measured equivalently in both arms. These differences describe the magnitude of the household-level comparison and are not the inferential basis of the study, for the reasons outlined in the Statistical Analysis section. Provincial-level values were analyzed using an exact Wilcoxon rank-sum test, with the province as the unit of observation. The smallest attainable two-sided p-value in a four-versus-16 configuration is 0.0004; a nonsignificant result at this level therefore reflects limited statistical power as much as the absence of a difference. ABC: activity-based contracting, CI: confidence interval, ITN: insecticide-treated net, RR: risk ratio

Indicator	Household-level risk difference, % (95% CI); risk ratio	Provincial-level: ABC (n = 4), median (range)	Provincial-level: Non-ABC (n = 16), median (range)	Exact p
Compliance with ITN allocation norms (%) - primary outcome	+8.8 (+8.6 to +9.0); RR 1.103 (1.100 to 1.105)	93.6 (91.8-98.9)	85.6 (75.1-91.5)	0.0004
Independently verified household coverage (%)	+2.8 (+2.7 to +2.9); RR 1.029 (1.028 to 1.030)	98.5 (97.5-99.9)	96.0 (92.0-99.9)	0.054
Adjusted campaign preparation time (weeks)	Not applicable at household level (measured per province)	14.5 (10.7-18.9)	19.1 (13.1-44.4)	0.083
Measured ITN loss rate (%)	Not tested (not measurement-equivalent between arms; see Statistical Analysis)	0.34 (0.26-0.37)	0.07 (<0.01-1.12)	0.060
Administrative coverage ratio (%)	Not tested (not a proportion; see Statistical Analysis)	94.2 (86.5-119.1)	92.7 (68.6-119.8)	0.682

Compliance with ITN allocation norms clearly distinguished the two groups at the provincial level: the four ABC provinces occupied the four highest ranks among the 20, and no non-ABC province reached the level of the lowest-performing ABC province (median 93.6% versus 85.6%; exact p = 0.0004, the smallest attainable value in this configuration). The same complete separation was found in the matched four-versus-four comparison (exact p = 0.029, again the smallest attainable value). None of the secondary outcomes reached statistical significance at the provincial level: independently verified coverage (p = 0.054), measured ITN loss rate (p = 0.060), adjusted campaign preparation time (p = 0.083), and the administrative coverage ratio (p = 0.682).

Figure 4 shows the household-level risk differences and 95% CIs for the two indicators amenable to this representation. Preparation time and cost were measured once per province rather than aggregated from household-level observations, and the loss rate and administrative coverage ratio were not measurement-equivalent or were not proportions, respectively; therefore, none of the four is represented on this figure, and their provincial-level comparisons in Table 8 constitute the appropriate summary for each.

**Figure 4:**
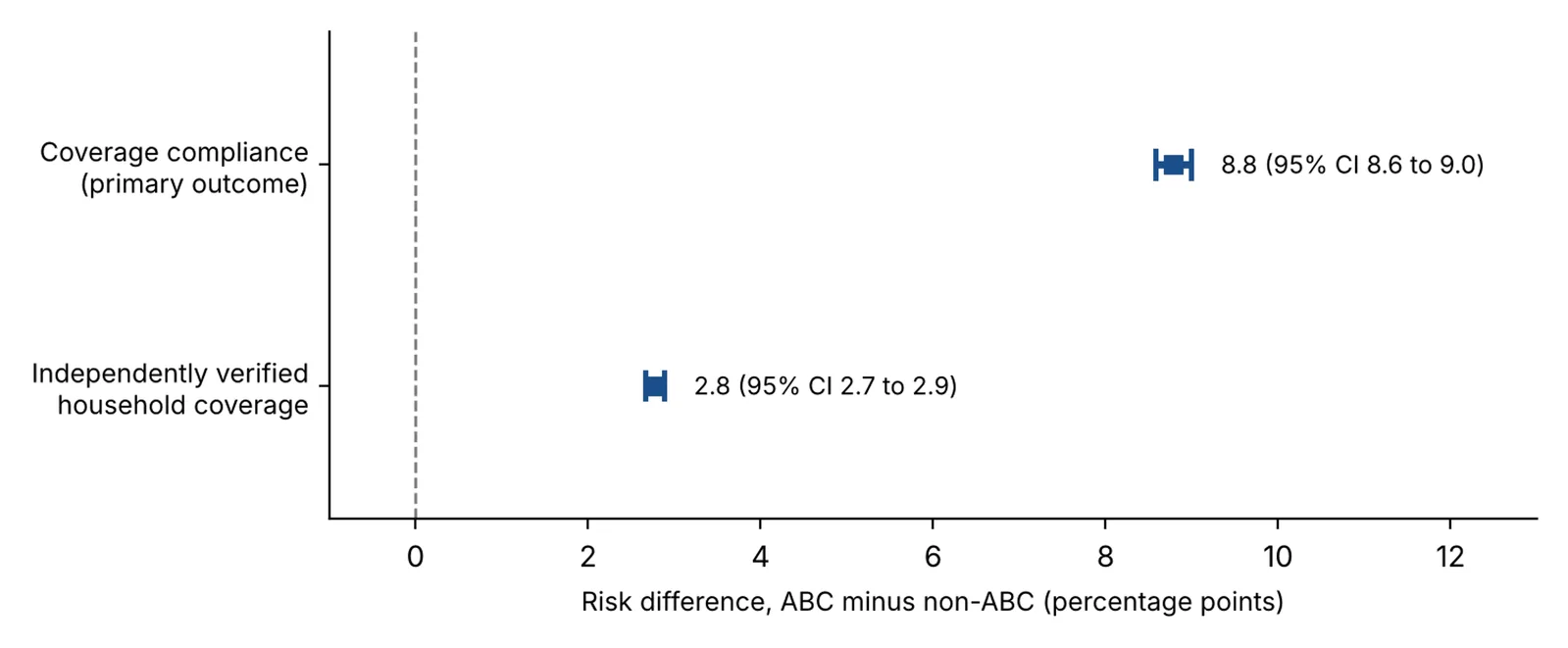
Household-level risk differences with 95% CIs for compliance with ITN allocation norms and independently verified household coverage (ABC versus non-ABC provinces) Squares indicate the point estimates of the risk differences (ABC minus non-ABC, in percentage points), and horizontal lines indicate the 95% CIs (calculated using Newcombe’s method, based on Wilson score intervals). The dashed vertical line indicates no difference. These intervals reflect the household-level denominator and are narrower than the true provincial-level uncertainty. CI: confidence interval, ITN: insecticide-treated net, ABC: activity-based contracting Image credit: Created by the SANRU team using Microsoft PowerPoint (Microsoft Corp., Redmond, Washington, USA).

## Discussion

The pilot ITN mass distribution campaign conducted in Kwilu suggested that the ABC approach could improve both the quantity and quality of household coverage and reduce campaign preparation time [12]. Extending the approach to three additional provinces allowed us to examine these preliminary findings against the heterogeneity of routine implementation.

Compliance with ITN allocation norms was consistently higher in provinces applying ABC across every comparison framework and, most notably, when the province rather than the household was the unit of analysis (Table 8). Independently verified household coverage, by contrast, was already close to the ceiling in both groups and did not differ significantly between them at the provincial level. Therefore, the intervention appears to be associated with improvements in coverage quality rather than volume.

This distinction is important programmatically. Household ownership of at least one ITN, long used as the standard coverage metric, rose from 5% to 70% in sub-Saharan Africa between 2000 and 2022 and from 9.2% in 2007 to 69.4% in 2023 in the DRC [4,15]. A more demanding indicator has since been proposed: the proportion of the population living in households with ITN access, defined as the number of people who could sleep under an ITN if each net were used by two people [16]. To operationalize this, the national malaria control program of the DRC allocates ITNs by household size: one ITN for one to two people, two for three to four, three for five to six, four for seven to eight, and five for nine or more [7]. With a mean household size of 5.2 people reported by the 2017-2018 Multiple Indicator Cluster Survey and only 9.2% of households containing 10 or more people according to the 2020 Sustainable Development Goals Indicator Cluster Survey [17,18], a ceiling of five ITNs per household is sufficient to optimize access. Conventional reporting records a household as covered if it receives fewer ITNs than its size requires, even though part of that household sleeps unprotected. The principal contractual deliverable is based on compliance with the allocation algorithm, counts toward 75% of the price payable to health zones [12], and is the dimension on which the present evaluation finds the clearest difference.

The direction of this result is consistent with evidence that financing conditioned on predefined contractual outcomes is associated with higher coverage of target populations [19], including an assessment of performance-based financing in the DRC that found improvements in structural quality, technical processes, and coverage of priority services relative to unconditional facility financing [19]. Such mechanisms are of particular interest at a time when progress against malaria has fallen behind the milestones of the Global Technical Strategy 2016-2030 and international financing has plateaued [3,20-23].

Measured ITN loss rates were higher under ABC than in the 16 non-ABC provinces (0.33% versus 0.17%), but the comparison with the same provinces' own preceding campaigns gives a more nuanced picture. The pooled rate differed by only 0.05 percentage points (0.33% versus 0.27%), and the direction was not uniform: losses were lower in Kwilu and Mongala and higher in Sud-Ubangi and Haut-Uélé, whose earlier campaigns had reported the lowest rates (0.06% and 0.08%). These figures require careful reading because the two approaches measure losses differently. Balances at the end of ABC campaigns are established by an independent physical count of remaining ITNs, carried out by a sub-recipient that assumes contractual liability for the quantity counted and subsequently distributes those ITNs through routine channels. Balances at the end of standard campaigns, including the earlier campaigns of the ABC provinces, are derived arithmetically from the difference between ITNs received and ITNs reported as distributed by community health workers. When the latter balances are later used for routine distribution, the quantities physically counted are generally lower than those previously reported, suggesting that the standard approach underestimates losses. The higher figures observed under ABC, particularly in Sud-Ubangi and Haut-Uélé, may therefore reflect more accurate measurement rather than poorer stock management. This interpretation does not explain the lower rates in Kwilu and Mongala, and because the two approaches were not measured equivalently, the available data cannot test it. Accordingly, loss rates are presented as descriptive data, and no inference is drawn from their comparison.

Within the ABC arm, where measurement is consistent, the loss data are informative in a different way. Beyond a contractual threshold of acceptable losses, set at below 1% during the Kwilu pilot and reduced to below 0.5% for the subsequent three provinces, all losses attract penalties deducted from the final payment to health zones. Losses above 0.5% are charged at the budgeted unit price of an ITN (USD 3.06) for destroyed but inventoried nets, at an amount equivalent to that price for uninventoried nets where the loss rate lies between 0.5% and 1%, and at twice that price where it exceeds 1%. Based on underlying behavioral models, such as the health belief model, we expected these penalties to deter subsequent provinces, in which perceived risk or consequence acts as a trigger for action [12,24]. Consistent with this, health zone management teams became more attentive to stock management, and the loss rate fell from 0.33% in Kwilu in June 2022 to 0.26% in Mongala in March 2023. Haut-Uélé, learning from penalties applied to earlier provinces, requested additional time to trace remaining ITNs before provincial validation of contractual deliverables, recovering 775 ITNs in the Gombari health zone and 1,327 in Wamba, and reducing its final loss rate from 0.52% to 0.37%.

However, despite an initial decline from 0.33% in Kwilu to 0.26% in Mongala, loss rates subsequently increased to 0.35% in Sud-Ubangi and 0.37% in Haut-Uélé. This pattern may reflect adaptation to the contractual terms, with arrangements calibrated to stay just below penalty thresholds and produce a non-negligible cumulative effect at the provincial level. Although this interpretation remains hypothetical, the pattern warrants continued monitoring and periodic recalibration of thresholds.

Unit costs were lower under ABC, at USD 0.668 per ITN distributed, compared to USD 0.773 under the standard approach in the same two provinces two to three years earlier. Applied to the 48,345,652 ITNs distributed under the standard approach between 2022 and 2024, a differential of this size would correspond to approximately USD 5.1 million. That figure is an order-of-magnitude illustration rather than an estimate, since it applies a differential observed in two provinces to ITNs distributed elsewhere under conditions that may not be comparable. Nonetheless, it indicates the scale of efficiency at stake under the seventh Global Fund grant cycle, in which a substantial funding gap for ITN mass campaigns has forced the prioritization of health zones in provinces previously covered in full. Of the 10 provinces planned for campaigns in 2025, North Kivu, South Kivu, and Kwango are targeted at 22.5%, 10.4%, and 76.5%, respectively, against 100% three years earlier.

The origin of these savings limits their generalizability. They appear to derive mainly from capping logistics costs at budgeted and contracted prices under ABC, whereas the standard approach retains a procurement process that, though lengthy, has sometimes secured lower prices in provinces with fewer logistical constraints, such as Haut-Katanga. Therefore, cost reductions achieved under ABC should not be extrapolated uniformly to all provinces. The reported unit costs also exclude the independent verification mechanism, which the principal recipient finances separately, so the cost advantage is only partially accounted for.

After adjusting for logistical delays, preparation time was 8.2 weeks shorter under ABC than in the 16 non-ABC provinces and six weeks shorter than in the same provinces’ earlier campaigns. Both differences lose statistical significance at the provincial level (p = 0.083) and are accordingly reported as exploratory rather than established effects.

These findings should be set against evidence questioning the efficiency of performance-based financing. Concerns have been raised regarding priority distortion and perverse incentives in low-income settings [25], equity risks arising where performance incentives disproportionately benefit facilities and areas that already perform well [26], and declining productivity over time in health centers implementing such schemes [27]. Our results align with this ambivalence rather than oppose it: the observed efficiency gain appears to derive largely from cost capping rather than behavioral change among implementers, and subsequent increase in loss rates across successive ABC campaigns is more consistent with adaptation to contractual thresholds than with sustained improvement. Both observations invite caution in interpreting short-term associations and underline the need for longitudinal evaluation of durability and equity.

The policy context is nonetheless moving in this direction. The Global Fund has issued guidance on integrating ABC into grant applications [28] and, in a November 2024 operational policy note, encouraged principal recipients to consider results-based financing as a means of increasing program effectiveness [29]. Its policy on challenging operating environments recommends flexibility, partnership, and innovation as levers for optimizing implementation [11]. Evidence of the kind presented here is directly relevant to countries operating under that policy, where the binding constraint on effective protection is often the quality rather than the volume of distribution. Whether the mechanism transfers to other campaign-based interventions and whether it holds once external supervision recedes remain open questions this evaluation cannot answer.

The comparison presented here cannot separate the effect of the contracting mechanism itself from other factors that may covary with it. Provinces receiving ABC also received concentrated technical assistance from SANRU and its sub-recipient, closer and more frequent supervision visits than the routine approach typically allows, and sustained engagement from the same donor-funded implementing partners across successive campaigns. Stronger local health zone management, more experienced supervisory staff, or simply greater donor and partner attention, independent of the contractual mechanism, could each produce findings similar to those reported here. The matched and historical comparisons narrow but do not eliminate this ambiguity, and the findings should be read as attributable to the ABC intervention package as a whole, of which contractual payment is one component among several that moved together.

These findings come from a single country’s malaria program and from provinces that, while contextually varied, do not represent all challenging operating environments in which the Global Fund recommends this type of flexibility [30]. Generalization beyond the DRC, to other diseases, or to other campaign-based interventions entirely should accordingly be treated with caution pending evaluation in other settings. Scaling ABC from four provinces to a fully national program would in addition raise implementation questions distinct from those addressed here: national scale-up would multiply the caseload of the independent verification agencies well beyond the 595,383 households visited in this evaluation, require contracting capacity across health zones with widely differing baseline management strength, and remove the concentrated partner attention that accompanied the four provinces evaluated. The present data do not address whether verification quality, contracting oversight, and the observed compliance gains would hold under that expansion.

Limitations

This study has several limitations worth considering. First, the comparative analysis relied on aggregated data from routine campaign reports and reports from independent verification agencies. While these sources are robust, they may be subject to reporting bias or inconsistent data-collection procedures across provinces.

Second, contextual heterogeneity among provinces (differences in population density, security conditions, and logistical infrastructure) may have influenced campaign performance independently of the contracting approach. Province-level malaria endemicity and formal health system capacity indicators were unavailable and thus unaddressed sources of heterogeneity, limiting the external validity of the comparison beyond the provinces studied. Although provinces were matched on administrative origin for the subgroup comparison, residual confounding cannot be excluded. The ABC assignment was not random. The possibility that provinces perceived as more likely to succeed under a contractual mechanism were preferentially selected cannot be excluded, potentially biasing the comparison in favor of ABC. Furthermore, factors accompanying ABC implementation, including more intensive supervision, sustained partner engagement, and stronger local management, may account for part of the observed association rather than the contractual mechanism itself. The within-province historical comparison mitigates but does not eliminate these concerns. The provincial-level analysis is also an ecological comparison. Each province is treated as a single observation, and the association observed at that level cannot be assumed to hold for any individual health zone or household within a province, a form of inference risk generally known as the ecological fallacy. Future studies should consider designs that better control for confounding, such as regression discontinuity or difference-in-differences analysis using health-zone-level rather than provincial-level data.

Third, the ABC approach estimated ITN loss rates through physical inventory verification, whereas the standard approach derived them from administrative records. This discrepancy limits comparability across approaches, and no statistical comparison of the two has been reported.

Fourth, within-province historical comparisons are susceptible to temporal confounding. Campaign design changed after the COVID-19 pandemic, notably by coupling household enumeration with same-visit distribution; logistical capacity and data-capture digitalization evolved over the period, and funding modalities shifted between Global Fund allocation cycles. Each of these would be expected to improve performance, regardless of the contracting approach, and their contribution cannot be separated from ABC's. Therefore, the historical comparison should be read as corroborating the concurrent comparison rather than as independent evidence. The historical compliance comparison is based on a single province.

Fifth, the statistical approach carries several limitations. Household-level comparisons relied on two-sample Z-tests for proportions applied to aggregated data, while the ABC approach was assigned at the provincial level; this mismatch overstates precision, and the resulting test statistics should not be read as measures of the strength of evidence. The provincial-level analysis was limited to four intervention provinces, where the smallest attainable two-sided p-value was 0.0004, and statistical power was correspondingly limited; absence of significance for the secondary outcomes should not be read as evidence of absence of effect. No multivariable adjustment or fixed-effects modeling was possible given the aggregated nature of the data. Because assignment was not randomized and the data are aggregated, the associations reported cannot be interpreted as causal effects of the contracting mechanism.

Sixth, this is a descriptive cost comparison, not a formal economic evaluation: no cost-effectiveness analysis relating expenditure to a health outcome, such as malaria cases or disability-adjusted life years averted, was performed, and the cost findings should be interpreted accordingly. The reported unit costs come from health zone micro-plan budgets and exclude the cost of operating the independent verification mechanism, which the principal recipient bears separately; therefore, the cost advantage attributed to ABC is only partial. The historical cost comparison (Table 6) also compares nominal USD budgets across campaigns conducted two to three years apart without adjustment for inflation or for movements in domestic prices for transport, fuel, and labor over that period, which may account for part of the observed difference independently of the contracting approach. A full economic evaluation incorporating verification costs, price-adjusted budgets, and a defined health outcome would be required before an efficiency claim could be considered established.

Seventh, the volume of records excluded during data cleaning cannot be quantified retrospectively because the routine campaign information system does not retain an audit trail of exclusions. Therefore, systematic differences in exclusion practices between provinces cannot be ruled out.

Eighth, the study did not assess longer-term outcomes such as sustained ITN use, malaria incidence, or cost-effectiveness beyond the campaign period. Longitudinal designs are required to evaluate the durability of any effect on malaria prevention.

Finally, the implementation team conducted this internal evaluation and is therefore subject to confirmation bias. Three features of the design limit, without eliminating, the resulting risk: the primary outcome and household coverage estimates were produced by independent third-party civil society verification agencies contractually distinct from the implementing team; results unfavorable to the ABC approach are reported without attenuation; and findings are triangulated across three comparison frameworks and a province-level reanalysis rather than resting on a single analysis. These measures do not substitute for external evaluation, which would be required before these findings could support policy recommendations beyond the DRC.

Despite these limitations, the study contributes real-world evidence on results-based contracting in large-scale public health interventions across diverse provincial contexts. The convergence of concurrent, historical, matched, and provincial-level analyses on the primary outcome, each subject to different threats to validity, strengthens confidence in this finding. In contrast, the divergence between household-level and provincial-level results for the secondary outcomes delimits what the data can and cannot support.

## Conclusions

In this multi-province evaluation, extending ABC beyond the Kwilu pilot was associated with higher compliance with ITN allocation norms (the proportion of households receiving the number of ITNs corresponding to their size) than the standard input-based approach. The association was consistent across the concurrent, historical, and matched comparison frameworks and persisted when the province, rather than the household, was the unit of analysis, with complete separation between the two groups.

Differences in independently verified household coverage and campaign preparation time favored ABC but did not reach statistical significance at the provincial level. Unit costs were also lower under ABC in the descriptive historical comparison; however, these findings remain exploratory. Measured ITN loss rates were higher under ABC, potentially reflecting more stringent physical verification rather than poorer stock management. This interpretation, however, could not be tested with the available data. The findings suggest that ABC may be a promising strategy for improving the quality of large-scale distribution campaigns in challenging operating environments and warrant further evaluation with more rigorous study designs.
